# Living Donor Versus Deceased Donor Pediatric Liver Transplantation: A Systematic Review and Meta-analysis

**DOI:** 10.1097/TXD.0000000000001219

**Published:** 2021-09-20

**Authors:** Arianna Barbetta, Chanté Butler, Sarah Barhouma, Rachel Hogen, Brittany Rocque, Cameron Goldbeck, Hannah Schilperoort, Glenda Meeberg, James Shapiro, Yong K. Kwon, Rohit Kohli, Juliet Emamaullee

**Affiliations:** 1 Department of Surgery, University of Southern California, Los Angeles, CA.; 2 Wilson Dental Library, USC Libraries, University of Southern California, Los Angeles, CA.; 3 Department of Surgery, University of Alberta, Edmonton, Canada.; 4 Department of Pediatrics, University of Southern California, Los Angeles, CA.; 5 Division of Gastroenterology, Hepatology and Nutrition, Children’s Hospital Los Angeles, Los Angeles, CA.; 6 Division of Abdominal Transplantation, Children’s Hospital Los Angeles, Los Angeles, CA.

## Abstract

**Background.:**

Reduced-size deceased donors and living donor liver transplantation (LDLT) can address the organ shortage for pediatric liver transplant candidates, but concerns regarding technical challenges and the risk of complications using these grafts have been raised. The aim of this study was to compare outcomes for pediatric LDLT and deceased donor liver transplantation (DDLT) via systematic review.

**Methods.:**

A systematic literature search was performed to identify studies reporting outcomes of pediatric (<18 y) LDLT and DDLT published between 2005 and 2019. A meta-analysis was conducted to examine peri- and postoperative outcomes using fixed- and random-effects models.

**Results.:**

Overall, 2518 abstracts were screened, and 10 studies met criteria for inclusion. In total, 1622 LDLT and 6326 DDLT pediatric patients from 4 continents were examined. LDLT resulted in superior patient survival when compared with DDLT at 1, 3, and 5 y post-LT (1-y hazard ratio: 0.58, 95% confidence interval [CI] 0.47-0.73, *P* < 0.0001). Similarly, LDLT resulted in superior graft survival at all time points post-LT when compared with DDLT (1-y hazard ratio: 0.56 [95% CI 0.46-0.68], *P* < 0.0001]. The OR for vascular complications was 0.73 (95% CI 0.39-1.39) and 1.31 (95% CI 0.92-1.86) for biliary complications in LDLT compared with DDLT, whereas LDLT was associated with lower rates of rejection (OR: 0.66 [95% CI 0.45-0.96], *P* = 0.03).

**Conclusions.:**

This meta-analysis demonstrates that LDLT may offer many advantages when compared with DDLT in children and suggests that LDLT should continue to be expanded to optimize outcomes for pediatric LT candidates.

## Abstract 2

Supplemental Digital Content is available in the text.

## Graphical abstract

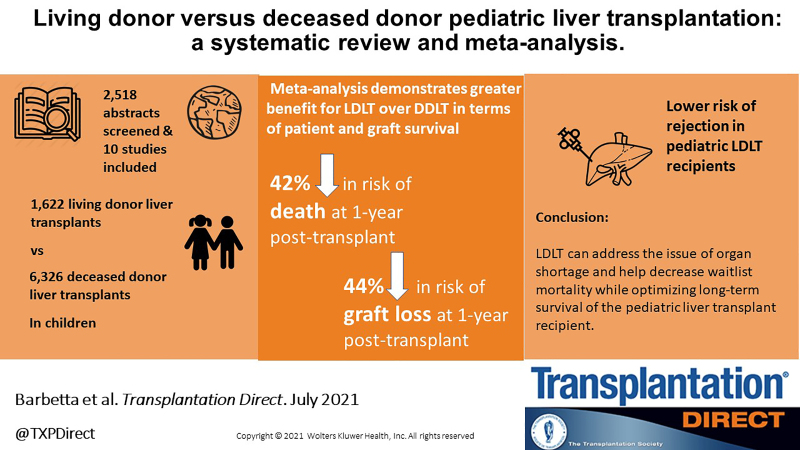

## INTRODUCTION

In children with acute and chronic liver disease requiring liver transplantation (LT), difficulty in identifying size-matched deceased donor organs continues to deepen organ shortage for pediatric recipients.^1,2^ In the United States, more than half of children listed for LT are <5 y old, and children <1 y of age experience the highest rates of pretransplant waitlist mortality.^1^ Expanded use of reduced-size or “split” deceased donor organs and living donor LT (LDLT) has reduced pediatric waitlist mortality.^3^ Even with the increased use of reduced-size grafts from deceased donors, these still represent <30% of all pediatric liver transplants performed in recent years in the United States.^4^ LDLT continues to represent an even smaller proportion of pediatric LT in the western world, with only 8.4% of pediatric candidates undergoing LDLT in the United States in 2018.^1^ Further considerable geographic variation exists in access to LDLT for American children.^5^

Although a high level of technical expertise and potential risks to a living donor may have limited the expansion of LDLT, there are still many advantages to this procedure.^6,7^ LDLT is elective, thereby reducing wait time and allowing for optimization, and as such, transplantation can occur before significant clinical deterioration, which often occurs more rapidly in children than adults.^6,8^ As the deceased donor pool trends toward more obese and marginal donors that may not be suitable for splitting, LDLT offers the potential for higher quality grafts with avoidance of steatosis, prevention of toxicity related to brain death, and shorter cold ischemic times.^2,6,7,9-12^

Data comparing pediatric deceased donor LT (DDLT) and LDLT overall outcomes are limited to retrospective database studies and small cohort studies, and heterogenous outcomes have been described.^2,6,11^ Some groups have reported equivalent survival following both DDLT and LDLT, whereas others suggest either DDLT or LDLT may be superior.^9,13-17^ The aim of this study was to compare outcomes in pediatric LDLT and DDLT recipients by conducting a systematic review and meta-analysis of studies reported in the last 15 y.

## MATERIALS AND METHODS

### Literature Search

According to the recommendations of the Preferred Reporting Items for Systematic Reviews and Meta-Analyses guidelines, the protocol for this systematic review was prospectively registered on the International Prospective Register of Systematic Reviews, PROSPERO (CRD42020164661). In collaboration with a health sciences librarian, a search strategy was developed, and a comprehensive search was conducted on the following databases on December 2, 2019: PubMed (coverage 1946–present), Embase and Embase Classic (coverage 1947–present), Cochrane Library (1898–present), Web of Science (coverage 1900–present), Clinicaltrials.gov, and Google Scholar (**Table S1, SDC**, http://links.lww.com/TXD/A364). A publication date filter for 2005–2019 was applied to capture the most recent 15 y of experience, and the systematic review was started in January 2020. No other filters were applied for study type, language, or any other limit. A combination of subject headings (when available) and keywords was used for the concepts “pediatrics,” “living donor,” “deceased donor,” and “liver transplantation.” Duplicated citations were removed in EndNote x9.2 using the Bramer method, and files were uploaded into Covidence for screening.^18^

### Study Selection

Title and abstract screening and full-text review were independently performed by 2 authors using the Covidence platform. All conflicts on study inclusion were resolved by the senior authors. The inclusion criteria were (1) prospective, retrospective cohort studies and randomized controlled trials designed to compare LDLT and DDLT; (2) age of transplant recipients <18 y; (3) studies published between January 2005 and December 2019; and (4) reporting the primary endpoint of patient survival at ≥1 y posttransplant. Studies were excluded if a full-text was not available, if the LDLT or DDLT cohort had <10 patients, or those lacking DDLT as a reference group. Studies including both adults and children, retransplants recipients, and/or multiple organ transplants were also excluded. To avoid data duplication, if 2 or more studies originated from the same center or included data from the same database, only the most recent publication or with the largest sample size or with more detailed data was included in the current meta-analysis. According to Cochrane Review guidelines and recommendations, unpublished data should be incorporated where possible to minimize bias.^19-22^ As the US registry data had recently been published and met inclusion criteria for this systematic review, we approached international centers with existing collaborative data-sharing agreements with our center. Not all centers maintain a detailed registry for their program, and due to limitations of the ongoing pandemic, only one additional international center was able to provide unpublished data for this review. Data were obtained from the University of Alberta in Canada, using the same inclusion/exclusion criteria, for transplants performed from January 2007 to December 2018, with ≥1 y of follow up. Combining these data with published reports from the University of Toronto enabled our review to capture the Canadian pediatric LT experience as there are no detailed registry reports available from the Canadian Organ Replacement Registry.^23^

### Data Extraction and Outcome Measures

Data concerning the design and study characteristics (first author, year of publication, country, study period) and patient cohort characteristics (sample size of the DDLT group, including numbers of technical variants such as split LT and reduced size volume (collectively referred to as “reduced size grafts”), size of LDLT group, patient demographics, Pediatric End-stage Liver Disease (PELD) score at LT, and cause of underlying liver disease were collected when available. The primary study outcomes were overall patient and graft survival. Secondary outcomes included preoperative variables (PELD and waiting time) and postoperative variables such as biliary complications (stricture, leak, and stenosis), vascular complications (hepatic artery, hepatic vein thrombosis, and portal vein thrombosis), acute cellular rejection (ACR), and infection.

### Quality Assessment

To assess the risk of bias, 4 authors analyzed the quality of each included study independently using the NIH Quality Assessment Tool for Case–Control Studies.^24^ The maximum total score on this scale was 12, and if no particularly worrisome bias were detected, studies were defined as good when scored no <9, fair (scored between 6 and 8), and poor (scored ≤5), otherwise the overall quality rating was assigned based on authors’ judgments (**Table S2, SDC**, http://links.lww.com/TXD/A364).

### Statistical Analysis

Descriptive statistics were used to summarize patient demographics and study characteristics; total number with percentage and mean with SD were used for categorical and continuous variables, respectively. When unavailable, mean and SD were estimated from the provided sample size, median, range, and/or interquartile range.^25,26^ All variables reported in ≥3 studies were pooled for analysis. Random effects model was applied to estimate both the odds ratio (OR) in case of categorical variables and mean difference (MD) in case of continuous variables. χ^2^ test, I^2^ were also used as measurement of studies heterogeneity. For survival analyses, the observed minus expected numbers of deaths/graft loss (O-E), and their variances were used to calculate individual hazard ratio (HR) and overall HR with a fixed-effect model. O-E and variances were estimated from other summary statistics such as Kaplan–Meier curves, *P* values, and number of total events (**Supplemental Methods S1, SDC**, http://links.lww.com/TXD/A364).^27,28^ SPSS v25 was used for descriptive statistics, and RevMan 5.3 was used to perform meta-analyses and generate forest plots. A *P* < 0.05 was considered statistically significant.

## RESULTS

### Systematic Review

**FIGURE 1.**
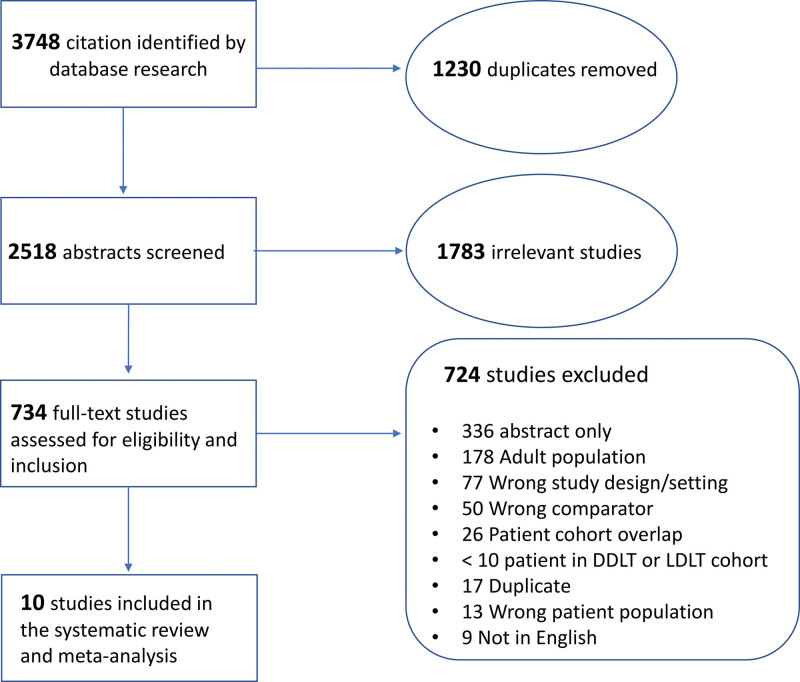
PRISMA diagram illustrating the results of systematic review process. DDLT, deceased donor liver transplantation; LDLT, living donor liver transplantation; PRISMA, Preferred Reporting Items for Systematic Reviews and Meta-Analyses.

Results of the complete literature search and review are summarized in the Preferred Reporting Items for Systematic Reviews and Meta-Analyses diagram (Figure 1). After removal of duplicated articles, a total of 2518 publications were screened by title and abstract, and 734 were selected for full-text review. Ten studies from China, Iran, Turkey, Belgium, Spain, Poland, Brazil, Canada, and the United States were identified for meta-analysis. Characteristics of the included studies are summarized in Table 1. All studies were retrospective, and one had a matched-paired design. Eight were from single centers, and 2 included multicenter data. No randomized control studies were included. According to the quality assessment evaluation, all articles were considered to have good or fair quality, none was deemed to be of poor quality (**Table S2, SDC**, http://links.lww.com/TXD/A364).

**TABLE 1.** Characteristics of included studies and patient population demographics.

Studies: author, year published, study period, country	Study design	Group	Sample size	Age, years (mean ± SD)	Sex, no. female (%)	PELD/MELD at transplant (mean ± SD)	Diagnosis (no.)								IS regimen
							^a^Cholestatic	Metabolic	Malignancy	Viral hepatitis	ALF	Cryptogenic	AIH	Other	
Aydogdu et al, 2005, 1997–2003, Turkey^29b^	Retrospective	LDLT	31	4 ± 3.6	13 (41.9%)	27 ± 12.3	14	17		4	5	11	6	5	Steroid + CNI
		DDLT	30	11 ± 4.4	19 (63.3%)	16 ± 13.3									
Oliveros et al., 2005, 1998–2005, Spain^13^	Matched cohort	LDLT	27	2.3			21	0	3		1	1		1	
		DDLT-whole	8	2.8			14	6	2		1	3		1	
		DDLT-partial	19												
Bahador et al. 2009, 1999–2008 Iran^57^	Retrospective	LDLT	54	9.1 ± 5.6		19.2 ± 12.9	31	41			8	23	20		Steroid + CNI + MMF
		DDLT-whole	64												
		DDLT-partial	20												
Leung Chan et al, 2009, 1993–2008 Hong Kong^30^	Retrospective	LDLT	59	3.0 ± 3.8	26 (44%)	20.8 ± 13.1	58	5	1	4	11				Steroid + CNI
		DDLT	19	3.6 ± 3.9	11 (57.9%)	18.3 ± 2.2									
Zhou et al, 2010, 1993–2009 China^16^	Multicenter retrospective	LDLT	208				118	125	23	7	14	5	3	24	Steroid + CNI and MMF Steroid + CNI
		DDLT-whole	72												
		DDLT-partial	46												
Darius et al, 2014, 1993–2010, Belgium^14b^	Retrospective	LDLT	203	1.1 ± 2.3	95 (46.8%)		153	12	21		2			15	Steroid + CNI + Aza, Steroid + CNI, Basiliximab + CNI
		DDLT-whole	88	3.3 ± 2.9	42 (47.7%)		64	11	0		3			10	
		DDLT-partial	138	2.1 ± 2.9	65 (47.1%)		93	11	6		18			10	
Tannuri et al, 2016, 1989–2014, Brazil^58^	Retrospective	LDLT	29								29				
		DDLT-Whole	10								50				
		DDLT-partial	40												
Szymczak et al, 2018, 1990–2016, Poland^59b^	Retrospective	LDLT	24	4 ± 3.7	16 (66.7%)	27.3 ± 23.6					24				
		DDLT-whole	31			36 ± 27.7					39				
		DDLT-partial	8												
University of Alberta, 2018, 2005–2017, Canada	Retrospective	LDLT	52	3.4 ± 4.2	25 (48.1%)	12.5 ± 16.1	29	11	6		6				
		DDLT-whole	29	6.6 ± 6.3	15 (51.7%)	10.4 ± 12.9	17	6	1		2		2	1	Basiliximab + CNI ± Steroid or MMF
		DDLT-partial	29	3.3 ± 3.7	16 (55.2%)	13.6 ± 13.5	20	5			3			1	
Montenovo, 2018, 2002–2016, USA^15^	Multicenter retrospective	LDLT	800	3.0 ± 4.7	408 (51%)	17.2 ± 13.8	502	70	43	7	107	19	8	44	
		DDLT-whole	3733	6.0 ± 6.1	1915 (51%)	13.9 ± 14.5	1598	610	363	37	546	176	123	280	
		DDLT-partial	1784	2.6 ± 3.7	893 (50%)	15.5 ± 14.9	924	237	183	4	263	66	17	90	
Kehar et al, 2019, 2000–2015, Canada^9b^	Retrospective	LDLT	135	1.1 ± 2.9	64 (47.4%)	10.7 ± 12.1	81	23	8		6			17	CNI ± MMF or Sirolimus ± MMF
		DDLT-whole	76	4.7 ± 7.7	72 (45.6%)	8.4 ± 11.4	55	29	12		35			27	
		DDLT-Partial	82												

^a^Biliary atresia, Alagille’s syndrome, primary sclerosing cholangitis, progressive familial intrahepatic cholestasis, Caroli disease.

^b^Denotes median to men conversion, calculated mean, or SD.

AIH, autoimmune hepatitis; ALF, acute liver failure; AZA, azathioprine; CNI, calcineurin inhibitor; DDLT, deceased donor liver transplantation; LDLT, living donor liver transplantation; MMF, mycophenolate mofetil; PELD, Pediatric End-stage Liver Disease.

### Meta-analysis

A total of 1570 LDLT and 6268 DDLT recipients were retrieved from published studies. Unpublished data from the University of Alberta were also included, resulting in 1622 LDLT and 6326 DDLT recipients being analyzed. Among DDLT recipients, 4111 children underwent whole LT, whereas 2166 received a reduced size graft. It was not possible to assess the exact proportion of deceased graft type in 2 of the included studies.^29,30^ The mean age of the entire patient cohort was 4.8 ± 5.5 y, with LDLT recipients transplanted at younger age (2.5 ± 4.2 y) when compared with all DDLT (4.8 ± 5.7 y, *P* < 0.0001 versus LDLT). Among the studies that reported DDLT subgroups, patients who received a reduced size graft were similar in age (2.6 ± 3.6 y) to LDLT (*P* = 0.77). Half of all LT recipients were female, and the most common indication for LT was cholestatic liver disease 47.7%, with a higher predominance in LDLT versus DDLT, (61.9% versus 45.8%, *P* < 0.001), followed by metabolic liver disease (15.3%), acute liver failure (14.8%), malignancy (8.5%), cryptogenic (3.8%), autoimmune hepatitis, (2.3%), and viral hepatitis (0.8%).

**FIGURE 2.**
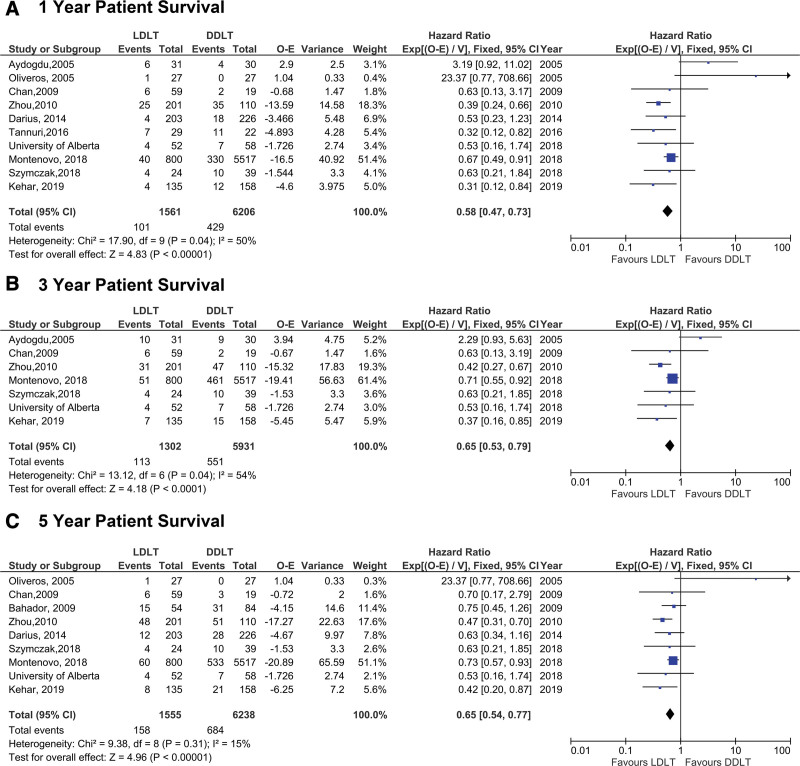
Comparison of overall survival (OS) between LDLT and DDLT recipients at (A) 1, (B) 3, and (C) 5 y post-LT. DDLT, deceased donor liver transplantation; LT, liver transplantation; O-E, observed minus expected numbers of deaths/graft loss.

The analysis of the primary outcome demonstrated superior overall patient survival in LDLT when compared with DDLT at all time points: 1, 3, and 5 y posttransplant (Figure 2). The HR of 0.58 across all studies represents a 42% reduction in hazard of death at 1 y post-LT for LDLT over DDLT recipients (95% confidence interval [CI] 0.47-0.73, *P* < 0.0001) (Figure 2A). The benefit of LDLT over DDLT on overall survival was also observed at 3- and 5-y overall survival post-LT (3 y HR: 0.65 [95% CI 0.53-0.79], *P* < 0.0001 and 5 y HR: 0.65 [95% CI 0.54-0.77], *P* < 0.0001, respectively] (Figure 2).

**FIGURE 3.**
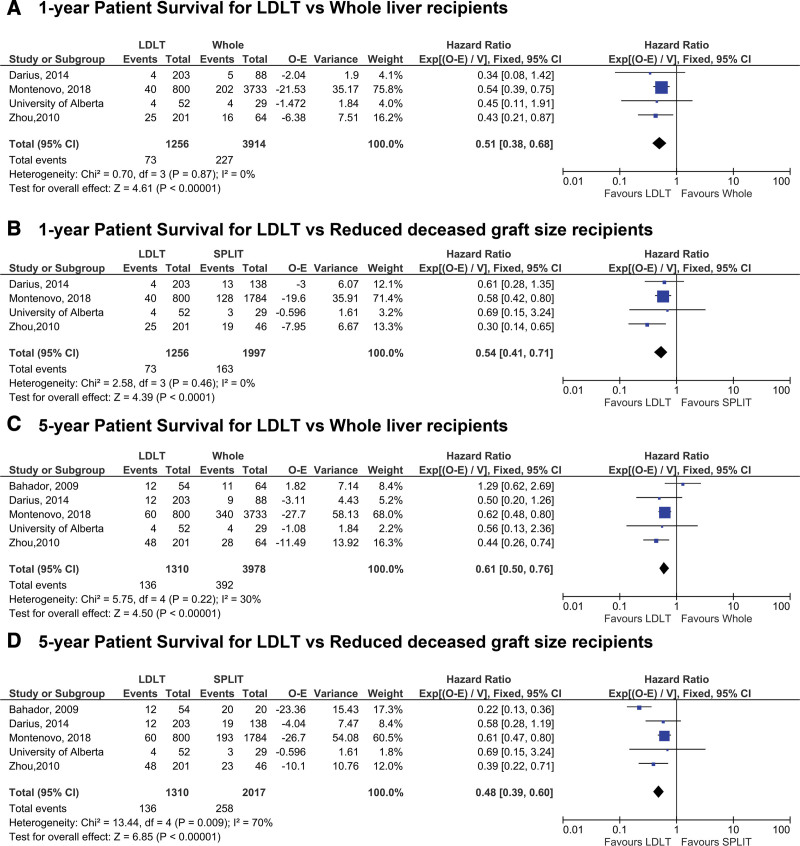
Patient survival stratified by deceased donor graft type at 1 y post-LT (panels A and B) and 5 y post-LT (panels C and D). LDLT, living donor liver transplantation; LT, liver transplantation; O-E, observed minus expected numbers of deaths/graft loss.

Subgroup analyses were conducted stratifying by type of deceased donor graft, demonstrating a superior 1-y overall survival in LDLT recipients when compared with whole liver recipients (HR: 0.51 [95% CI 0.38-0.68], *P* < 0.0001) as well as reduced size graft recipients (HR: 0.54 [95% CI 0.41-0.71], *P* < 0.0001) (Figure 3A and B). A similar result was observed at 5-y post-LT for overall survival after LDLT versus both whole liver and reduced graft recipients (HR: 0.61 [95% CI 0.50-0.76], *P* < 0.0001 and HR:0.48 [95% CI 0.39-0.60], *P* < 0.0001) (Figure 3C and D). Similar to patient survival, we found modest heterogeneity, most likely driven by the single outlying Oliveros study.

**FIGURE 4.**
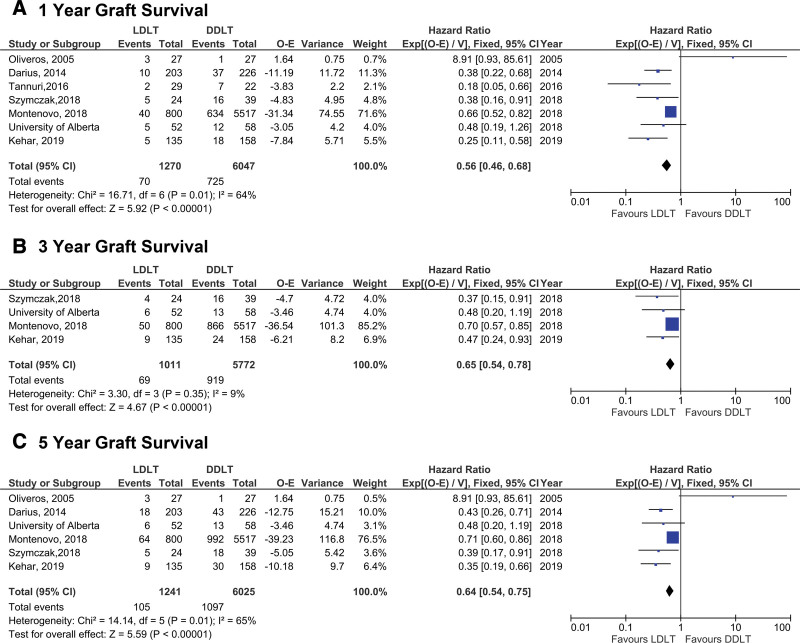
Comparison of graft survival between LDLT and DDLT recipients at (A) 1, (B) 3, (C) 5 y post-LT. DDLT, deceased donor liver transplantation; LDLT, living donor liver transplantation; LT, liver transplantation; O-E, observed minus expected numbers of deaths/graft loss.

Assessment of the second primary outcome, graft survival, indicated that LDLT recipients experienced superior graft survival at 1 y posttransplant when compared with DDLT recipients (HR: 0.56 [95% CI 0.46-0.68], *P* < 0.0001) (Figure 4A). Similar results were observed at both 3 and 5 y post-LT (HR: 0.65 [95% CI 0.54-0.78], *P* < 0.0001 and HR: 0.64 [95% CI 0.54-0.75], *P* < 0.0001) (Figure 4B and C). A subgroup analysis assessing graft survival between LDLT and either whole or reduced size deceased donor recipients also showed superior graft survival following LDLT (*P* < 0.0001) (**Figure S1, SDC**, http://links.lww.com/TXD/A364).

To measure the robustness of the findings and assess how the US registry study, which contributed the greatest number of patients and thus has the potential to skew meta-analysis results, could have influenced patient or graft survival, sensitivity analyses were performed by excluding it. Using this approach, the overall HR direction did not change for patient mortality or graft failure. Children who received a living donor graft had still a lower risk of death and graft failure at 1, 3, and 5 y post-LT, suggesting the US registry study does not obscure findings from the other regions (**Figures S2 and S3, SDC**, http://links.lww.com/TXD/A364).

**FIGURE 5.**
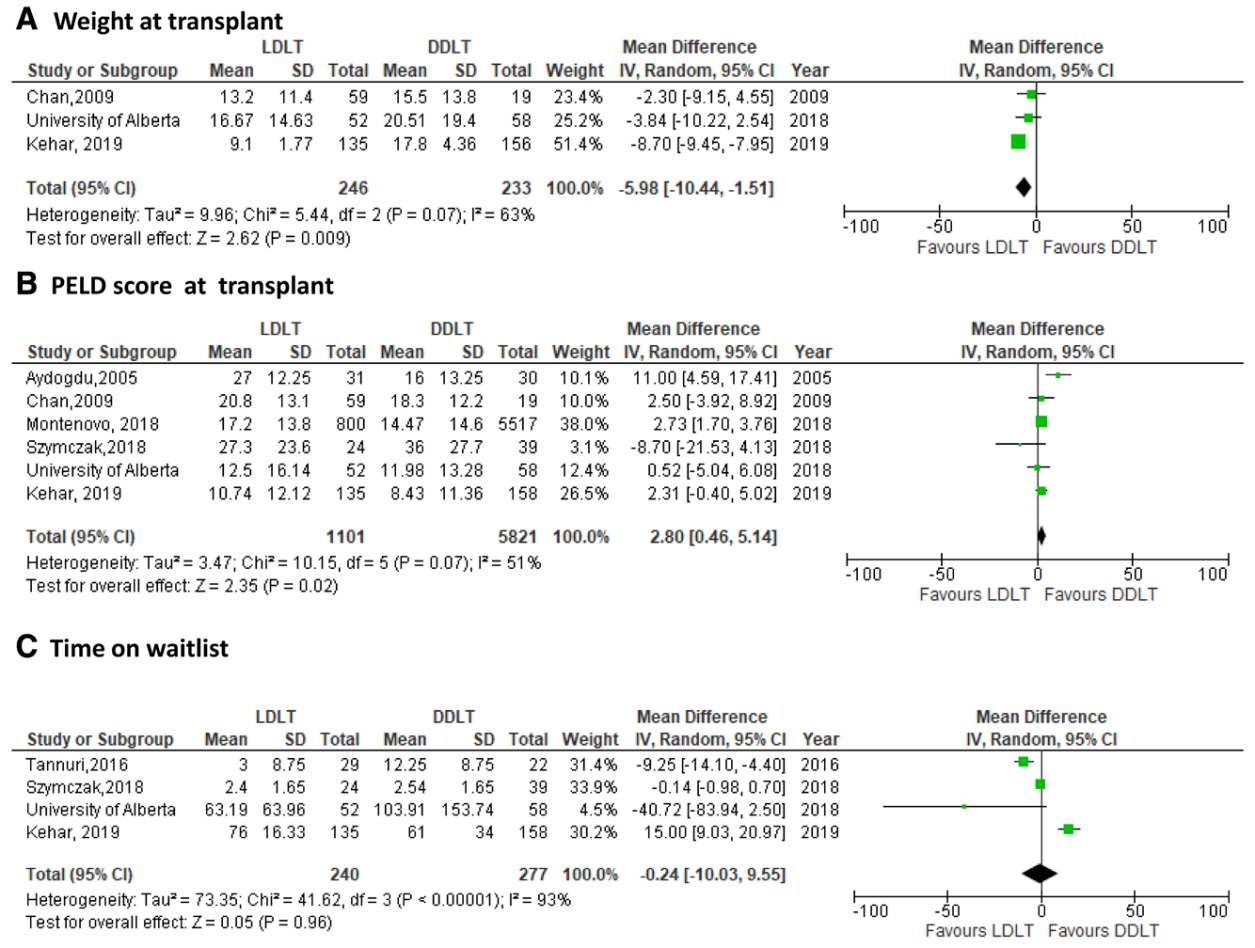
Forest plot of preoperative variables: (A) weight at LT, (B) PELD score, and (C) time on waitlist. DDLT, deceased donor liver transplantation; LDLT, living donor liver transplantation; LT, liver transplantation; PELD, Pediatric End-stage Liver Disease.

Three preoperative variables, weight, PELD score at LT, and time on waitlist were assessed as secondary outcomes (Figure 5). We observed a high degree of heterogeneity across these outcomes with I^2^ = 93% for time on waitlist; there were also fewer studies in each analysis and therefore more uncertainty in our estimates. As shown in Figure 5A, LDLT recipients had lower weight (assessed in kg) at LT when compared to DDLT (MD: 5.98 [95% CI −10.44 to 1.51], *P* = 0.009). Although PELD score at LT was higher in LDLT recipients than DDLT (MD: 2.80 [95% CI 0.46-5.14], *P* = 0.02) (Figure 5B), there was no difference in days spent on waiting list (MD: −0.24 [95% CI −10.03 to 9.53], *P* = 0.96) (Figure 5C). The number of studies with these outcomes were limited and more sensitive to choice of estimation method. We conducted the same analysis in R using Hartung–Knapp–Sidik–Jonkman method, opposed to the DerSimonian–Laird in RevMan. Although point estimates broadly remained the same, CIs did widen (results not shown), highlighting sensitivity in this calculation.

**FIGURE 6.**
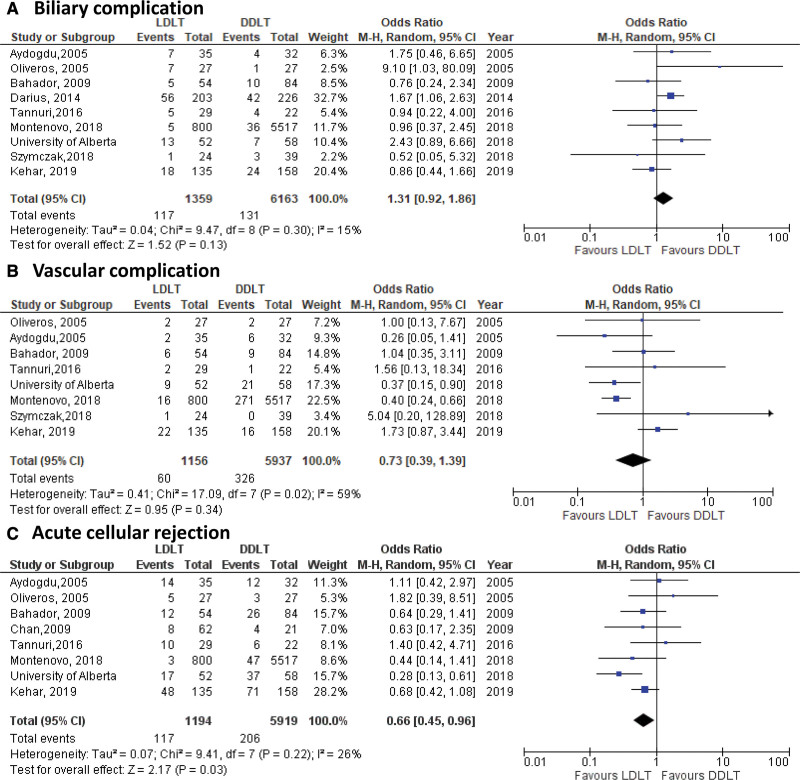
Forest plots of postoperative complications: (A) biliary complications, (B) vascular complication, and (C) acute cellular rejection. DDLT, deceased donor liver transplantation; LDLT, living donor liver transplantation.

Assessment of 2 postoperative technical outcomes showed an OR of 0.73 (95% CI 0.39-1.39, *P* = 0.34) for vascular complications and OR of 1.31 (95% CI 0.92-1.86, *P* = 0.13) for biliary complication in LDLT compared with DDLT recipients (Figure 6A and B). Furthermore, no differences in the rate of biliary complications were observed when analyses were stratified by deceased graft type (results not shown). Finally, pooled analysis for the odds of ACR demonstrated a lower risk of rejection in LDLT recipients when compared with DDLT (OR: 0.66 [95% CI 0.45-0.96], *P* = 0.03) (Figure 6C).

## DISCUSSION

As a consequence of limited access to deceased donors for both cultural and religious reasons, LDLT is the dominant approach to pediatric LT in the Middle East and Asia.^31-33^ Despite the existence of >25 pediatric liver transplant centers with experience in LDLT in the United States, low rates of LDLT utilization persist. In 2018, LDLT was reported in only 19 US states, with the majority of LDLT cases occurring in 5 centers.^5^ Over the same period, 7% of pediatric LT candidates in the United States were removed from the waiting list for medical deterioration or death, the highest rate being observed among those patients <1 y of age.^1^ Although excellent outcomes can be achieved following DDLT in children, this meta-analysis demonstrates clear benefit for LDLT over DDLT in terms of patient and graft survival at all time points: 1, 3, and 5 y posttransplant. Even with further stratification by deceased graft type including reduced size grafts, LDLT resulted in superior overall patient survival and graft survival at all time points.

Although graft survival was superior in the LDLT population when compared with DDLT (including both whole and reduced size grafts), there was no difference in the odds of vascular and biliary complications. Our observation of equivalent, generally low rates of technical complications following both LDLT and DDLT in children suggests that centers have gained experience, thus overcoming many of the technical hurdles that can affect post-LDLT outcomes.^34,35^ Indeed, a study published in 2007 from the Studies in Pediatric Liver Transplantation consortium reported decreased graft and patient survival, as well as increased 30-d postoperative morbidity, when technical variant grafts, including LDLT, were compared with whole organs.^36^ A more recent US registry analysis demonstrated that although long-term overall survival following LDLT was similar to the outcome of whole graft recipients in an early era (2002–2009), LDLT and reduced-size grafts resulted in better overall survival compared with whole grafts in the recent era 2010–2015.^4^ These observations were attributed to progressive experience and improved technique in recent years, which is supported by this meta-analysis, where the majority of patient data were reported in the last 10 y.

When examining secondary outcomes, LDLT was associated with a lower rate of ACR when compared with DDLT recipients. This may represent a major factor contributing to the superior graft survival following pediatric LDLT observed in this meta-analysis. Graft longevity is particularly important in the pediatric transplant population, as subclinical, histological evidence of inflammation and fibrosis are ubiquitous after <2 decades post-LT.^37,38^ It has been demonstrated that pediatric LDLT may offer immunologic advantages as it has been associated with a lower rate of ACR, chronic rejection, and can require less immunosuppression when compared to DDLT.^39-42^ Moreover, there are increasing data suggesting that maternal donor allografts may offer immunologic advantages that translate to a lower risk of ACR and improved ACR-free survival, possibly due to maternal–fetal microchimerism.^43^ Unfortunately, given the limitations of data available, we were not able to investigate donor and recipient relationships in the LDLT cohort to further explore this concept.

In this meta-analysis, we observed that LDLT recipients were transplanted with a higher PELD than DDLT recipients. It is also interesting to note that even though PELD was higher in the LDLT cohort, these patients experienced better survival at all time points in this meta-analysis. A recent analysis of US registry data showed that the estimated 90-d mortality risk using the PELD score underestimated the true pre-LT mortality in children by as much as 17%.^44^ Also, PELD may not capture the severity of disease in children with acute and chronic liver failure, which may be related to the fact that renal dysfunction is not captured by PELD.^45^ It is possible that PELD is higher in the pediatric LDLT population as parents/guardians of the sickest LT candidates may develop a sense of urgency to pursue LDLT as their child’s clinical condition deteriorates on the waiting list.

In this analysis, only a subset of analyzed studies reported waiting time, but in these studies, we did not observe any difference between LDLT and DDLT. Wait time is clearly a complex variable and likely relates to access to high-quality deceased donor organs. In the United States, adult LDLT recipients experience longer waiting times when compared with DDLT, which may be related to a sense of urgency to find a suitable living donor after an extended waiting time with seemingly no progress.^46^ Any strategy that can reduce waiting time for pediatric LT candidates should be pursued. Delays in LT for pediatric candidates not only contributes to a higher degree of mortality but also contributes to exacerbations in growth and cognitive delays for those who ultimately undergo transplantation.^4,47^ Transplantation with a shorter waiting time can avoid developmental impairment by reducing frequency of hospitalization, progressive malnutrition, and growth failure before LT, thereby leading to better functional outcomes, especially among small children.^8,48^ Given the findings of this meta-analysis, early LDLT in the pediatric liver transplant recipient should prevent waitlist morbidity.

Countries with a higher proportion of pediatric LDLT and split DDLT have decreased waitlist mortality, which is likely multifactorial and the result of each national healthcare system’s ability to provide access to high-level transplant care.^49^ In the United States, pediatric waitlist mortality is reported to be around 8%–12% per year with a median waiting time of 100 d, with patients <1 y of age experiencing a disproportionately high rate of morbidity and mortality.^49-51^ Although splitting a deceased donor liver can improve access to size-matched allografts for pediatric candidates, 2 recent analyses of US data illustrated that only a small percentage ranging from 3.4% to 3.8% of “splittable” deceased donor grafts were actually split.^3,52^ Even with new allocation policies, the logistics of having a procurement surgeon with appropriate technical expertise, performing technically demanding surgery at offsite facilities, and appropriately allocating the remaining right trisegment graft are all major barriers in practice.

This meta-analysis supports the concept of expanded use of LDLT as an additional mechanism to address the issue of organ shortage and decrease waitlist morbidity and mortality in children. There are also emerging data that allocating nondirected, anonymous living donors towards pediatric candidates offers these donors a low morbidity procedure via left lateral segment donation and results in excellent long-term outcomes for pediatric recipients.^53,54^

There are limitations to this study. By design, we required that eligible studies compared LDLT and DDLT, thus studies from centers that exclusively performed either LDLT or DDLT were not included; however, we believe this allowed us to balance other potential confounders such as center specific volume, training, patient demographics, and clinical skills. Specifically, our literature search strategy and study design did not identify any studies from high-volume LDLT countries such as South Korea, Japan, and India that studied both pediatric DDLT and LDLT outcomes simultaneously. Also, although the eleven studies included in this meta-analysis represent 4 continents, the US data represents >50% of the LDLT and DDLT cohorts, which may have impacted some of the results. Further, we only found retrospective studies to analyze, as no randomized controlled trials were available that matched our inclusion criteria. Additionally, to increase the quality, data were screened by center to exclude studies with potential overlapping patient cohorts. However, not all the included studies reported data on graft survival or each of the secondary outcomes, and additionally, some a priori established variables (hospitalization status pre-LT, length of stay, post-op infection, donor relationship, ABO compatibility) were not widely reported. Although the immunosuppression regimens were described for the overall patient cohort in 7 of 11 studies, none of them compared this variable between recipients of DDLT versus LDLT, which represents a possible source of bias. Also, study heterogeneity, reflecting the differences in practice, policies, and ethics, may lead to selection bias and possibly be reflected in the outcomes studied as well.

There are also limitations in the statistical methods used. Bias, skew, or imbalance presented in any study will influence the findings in the meta-analysis; although in many cases, this is related to small sample size, and therefore, studies are accordingly weighted less. Additionally, calculations make normality assumptions for mean, SD, and CI estimation, which may not perfectly reflect the data and introduce possible bias for data with non-normal distribution. The use of fixed effects models may also produce overly narrow CIs depending if substantial heterogeneity is present. RevMan also provides technical limitations in available estimation techniques that may be optimal for the data.

Lastly, per our study design, some factors were not considered, such as the recurrence of disease and its impact on patient outcome.

In the adult population, the advantages of LDLT have been described in terms of improved overall patient survival, improved graft survival, transplant at a lower MELD, and decreased resource utilization.^55,56^ This study offers a broad and global view highlighting several beneficial effects related to LDLT in children. Existing single-center and registry study data have reported heterogeneous outcomes for patient and graft survival among pediatric LT recipients. We have demonstrated through systematic review of worldwide data, including both lower- and higher-volume centers and with a large pooled number of patients for both LDLT and DDLT groups, that LDLT recipients, despite having a higher PELD score at transplant, had improved graft and patient survival, as well as a lower rate of ACR posttransplant. Moreover, contrary to what has been reported so far, the risk of postoperative technical complications is similar between DDLT and LDLT in children. Based on our analysis, we propose that LDLT is one strategy that may address the critical issues of organ shortage and help decrease waitlist mortality while optimizing long-term survival of the pediatric liver transplant recipient.

## Supplementary Material


