# Supplementary material for: Living Donor Versus Deceased Donor Pediatric Liver Transplantation: A Systematic Review and Meta-analysis
Source: Transplant Direct. 2021 Sep 20;7(10):e767. doi: 10.1097/TXD.0000000000001219 (PMC8454909; doi:10.1097/TXD.0000000000001219)
Supplement: Supplementary file 1 [file txd-7-e767-s001.pdf]

**Database Name**<br>***Database Vendor***<br>*Dates of coverage*	**Search strategy**<br>Publication date filter: 2005-2019
**PubMed**<br>***U.S. National Library of Medicine***<br>*1946 – Present*	((Infant[Mesh] OR Child[Mesh] OR Adolescent[Mesh] OR Minors[Mesh] OR Pediatrics[Mesh] OR postnatal OR newborn OR newborns OR infant OR infants OR baby OR babies OR toddler OR toddlers OR child OR children OR kid OR kids OR juvenile OR juveniles OR youth OR youths OR young OR girl OR girls OR boy OR boys OR preadolescent OR preadolescents OR pre-adolescent OR pre-adolescents OR preteen OR preteens OR pre-teen OR pre-teens OR puberty OR prepuberty OR pubescent OR prepubescent OR adolescent OR adolescents OR teen OR teens OR teenager OR teenagers OR pediatric OR pediatrics OR paediatric OR paediatrics)<br><br>AND<br><br>(((live OR living) AND (donor OR donors OR donation OR "Tissue Donors"[Mesh])) OR "Living Donors"[Mesh])<br><br>AND<br><br>((deceased OR cadaveric OR cadaver OR "Cadaver"[Mesh]) AND (donor OR donors OR donation OR "Tissue Donors"[Mesh]))<br><br>AND<br><br>(((liver OR hepatic OR "Liver"[Mesh]) AND (transplant* OR graft* OR allograft* OR allotransplant* OR homotransplant* OR hepatectomy OR hepatectomies OR resection* OR lobectomy OR lobectomies OR segmentectom* OR "Transplantation"[Mesh] OR "Transplants"[Mesh] OR "Hepatectomy"[Mesh])) OR "Liver Transplantation"[Mesh]))<br><br>OR<br><br>(LDLT AND DDLT)
**Embase & Embase Classic**<br>***Elsevier***<br>*1947 – Present*	(('minor (person)'/exp OR 'pediatrics'/exp OR 'juvenile'/exp OR 'adolescence'/exp OR postnatal OR newborn OR newborns OR infant OR infants OR baby OR babies OR toddler OR toddlers OR child OR children OR kid OR kids OR juvenile OR juveniles OR youth OR youths OR young OR girl OR girls OR boy OR boys OR preadolescent OR preadolescents OR preadolescent OR pre-adolescents OR preteen OR preteens OR pre-teen OR pre-teens OR puberty OR prepuberty OR pubescent OR prepubescent OR adolescent OR adolescents OR teen OR teens OR teenager OR teenagers OR pediatric OR pediatrics OR paediatric OR paediatrics)<br><br>AND<br><br>(((live OR living) AND (donor OR donors OR donation OR 'donor'/exp)) OR 'living donor'/exp)<br><br>AND<br><br>(((deceased OR cadaveric OR cadaver OR 'cadaver'/exp) AND (donor OR donors OR donation OR 'donor'/exp)) OR 'cadaver donor'/exp)<br><br>AND<br><br>(((liver OR hepatic OR 'liver'/exp) AND (transplant* OR graft* OR allograft* OR allotransplant* OR homotransplant* OR hepatectomy OR hepatectomies OR resection* OR lobectomy OR lobectomies OR segmentectomy OR segmentectomies OR 'transplantation'/exp OR 'lobectomy'/exp OR 'segmentectomy'/exp)) OR ('liver transplantation'/exp OR 'liver resection'/exp)))<br><br>OR<br><br>(LDLT AND DDLT)
**Cochrane Library**<br>***Wiley***<br>*Cochrane Database of Systematic Reviews*<br>*(1995–present)*<br>*Cochrane Central Register of Controlled Trials*<br>*(1898–present)*	#1 live<br>#2 living<br>#3 #1 or #2<br>#4 donor donors<br>#5 donation<br>#6 MeSH descriptor: [Tissue Donors] explode all trees<br>#7 #4 or #5 or #6 or #7<br>#8

	#9	#3 and #8
	#10	MeSH descriptor: [Living Donors] explode all trees
	#11	#9 or #10
	#12	deceased
	#13	cadaveric
	#14	cadaver
	#15	MeSH descriptor: [Cadaver] explode all trees
	#16	#12 or #13 or 14 or #15
	#17	#16 and #8
	#18	liver
	#19	hepatic
	#20	MeSH descriptor: [Liver] explode all trees
	#21	#18 or #19 or #20
	#22	transplant*
	#23	graft*
	#24	allograft*
	#25	allotransplant*

	#26	homotransplant*
	#27	hepatectomy
	#28	hepatectomies
	#29	resection*
	#30	lobectomy
	#31	lobectomies
	#32	segmentectom*
	#33	MeSH descriptor: [Transplantation] explode all trees
	#34	MeSH descriptor: [Transplants] explode all trees
	#35	MeSH descriptor: [Hepatectomy] explode all trees
	#36	#22 or #23 or #24 or #25 or #26 or #27 or #28 or #29 or #30 or #31 or #32 or #33 or #34 or #35
	#37	#21 and #36

	#38	MeSH descriptor: [Liver Transplantation] explode all trees
	#39	#37 or #38
	#40	MeSH descriptor: [Infant] explode all trees
	#41	MeSH descriptor: [Child] explode all trees
	#42	MeSH descriptor: [Adolescent] explode all trees
	#43	MeSH descriptor: [Minors] explode all trees
	#44	MeSH descriptor: [Pediatrics] explode all trees
	#45	postnatal
	#46	newborn
	#47	newborns
	#48	infant
	#49	infants
	#50	baby
	#51	babies
	#52	toddler
	#53	toddlers
	#54	child
	#55	children
	#56	kid
	#57	kids
	#58	juvenile
	#59	juveniles
	#60	youth
	#61	youths
	#62	young
	#63	girl
	#64	girls
	#65	boy
	#66	boys
	#67	preadolescent

	#68 preadolescents<br><br>#69 pre-adolescent<br><br>#70 pre-adolescents<br><br>#71 preteen<br><br>#72 preteens<br><br>#73 pre-teen<br><br>#74 pre-teens<br><br>#75 puberty<br><br>#76 prepuberty<br><br>#77 pubescent<br><br>#78 prepubescent<br><br>#79 adolescent<br><br>#80 adolescents<br><br>#81 teen<br><br>#82 teens<br><br>#83 teenager<br><br>#84 teenagers<br><br>#85 pediatric<br><br>#86 pediatrics<br><br>#87 paediatric<br><br>#88 paediatrics<br><br>#89 #40 or #41 or #42 or #43 or #44 or #45 or #46 or #47 or #48 or #49 or #50 or #51 or #52 or #53 or #54 or #55 or #56 or #57 or #58 or #59 or #60 or #61 or #62 or #63 or #64 or #65 or #66 or #67 or #68 or #69 or #70 or #71 or #72 or #73 or #74 or #75 or #76 or #77 or #78 or #79 or #80 or #81 or #82 or #83 or #84 or #85 or #86 or #87 or #88 #90 #11 and #17 and #39 and #89<br>#91 LDLT<br><br>#92 DDLT<br><br>#93 #91 and #92<br><br>#94 #90 or #93
**Web of Science Core Collection**<br>***Clarivate Analytics***<br>*Science Citation Index Expanded (1900-present)*<br>*Social Sciences Citation Index (1900-present)*<br>*Arts & Humanities Citation Index (1975-present)*	((postnatal OR newborn OR newborns OR infant OR infants OR baby OR babies OR toddler OR toddlers OR child OR children OR kid OR kids OR juvenile OR juveniles OR youth OR youths OR young OR girl OR girls OR boy OR boys OR preadolescent OR preadolescents OR pre-adolescent OR pre-adolescents OR preteen OR preteens OR pre-teen OR preteens OR puberty OR prepuberty OR pubescent OR prepubescent OR adolescent OR adolescents OR teen OR teens OR teenager OR teenagers OR pediatric OR pediatrics OR paediatric OR paediatrics)<br><br>AND<br><br>((live OR living) AND (donor OR donors OR donation))

Conference Proceedings Citation Index:<br>*Science (1990-present)*<br>*Social Science & Humanities (1990-present)*<br>*Book Citation Index:*<br>*Science (2005-present)*<br>*Social Sciences & Humanities (2005-present)*<br>*Emerging Sources Citation Index (2015-present)*<br>*Current Chemical Reactions (1985-present)*<br>*Index Chemicus (1993present)*	AND<br><br>((deceased OR cadaveric OR cadaver) AND (donor OR donors OR donation))<br><br>AND<br><br>((liver OR hepatic) AND (transplant* OR graft* OR allograft* OR allotransplant* OR homotransplant* OR hepatectomy OR hepatectomies OR resection* OR lobectomy OR lobectomies OR segmentectomy OR segmentectomies)))<br><br>OR<br><br>(LDLT AND DDLT)
**Clinicaltrials.gov**<br>***U.S. National Library of Medicine***	Advanced search<br><br>Child (birth-17) filter<br><br>Intervention box: ((live OR living) OR (deceased OR cadaveric OR cadaver)) AND ((donor OR donors OR donation) AND (liver OR hepatic) AND (transplant OR transplantation OR graft))
**Google Scholar**	(child OR pediatric OR paediatric) (live OR living) (deceased OR cadaveric OR cadaver) (donor OR donors OR donation) (liver OR hepatic) (transplant OR transplantation OR graft)

Supplemental Table S1: Search Strategy. Date Last Searched: December 2, 2019.

Author Year	1. Research Question	2. Study Population	3. Sample Size Justification	4. Groups recruited from the same population	5. Inclusion/ Exclusion Criteria	6. Differentiation Between Cases and Controls	7. Random Selection	8. Use of Concurrent Controls	9. Exposure assessed prior to outcome measurement	10. Exposure measures and assessment	11. Blinding of exposure assessors	12. Statistical analysis	Total Score (out of 12)	Quality Rating (Good, Fair, or Poor)
	Yes (1), No(0), Other (CD, NR, NA)* "*CD, cannot determine; NA, not applicable; NR, not reported"													
Aydogdu 2005	1	1	0	1	1	1	NA	1	1	1	0	0	8	Fair
Bahador 2009	0	1	0	1	1	1	NA	1	1	1	0	0	7	Fair
Chan 2009	1	1	0	1	1	1	NA	1	1	1	0	0	8	Fair
Darius 2014	1	1	0	1	1	1	NA	1	1	1	0	1	9	Good
Kehar 2019	1	1	0	1	1	1	NA	1	1	1	0	1	9	Good
Montenovo 2019	1	1	0	1	1	1	NA	1	1	1	0	1	9	Good
Oliveros 2005	1	1	0	1	1	1	NA	1	1	1	0	0	8	Fair
Szymczak 2018	1	1	0	1	1	1	NA	1	1	1	0	0	8	Fair
Tannuri 2016	1	1	0	1	1	1	NA	1	1	1	0	1	9	Good
Zhou 2010	1	1	0	1	1	1	NA	1	1	1	0	1	9	Good

Supplemental table S2: Quality Assessment Table

Supplemental methods S1:

When number of events or percentage were not directly presented in the study, data for patient and graft survival were extracted from Kaplan Meier curves using the Webplotdigitizer (https://automeris.io/WebPlotDigitizer/). Then, Observed minus Expected number of cases was calculated applying the following formulas:

&'()*+),)+)./(*)()0*ℎ 0*2 × &'()*+),)+)./(3&./*&4 0*2
! − # = % × (7 (3&*) 8&* 9 +04:): 2)
/&/04 &'()*+),)+)./(

Variance was calculated as: "= ! "#$%&$' $&$)*# %$#$+%,-
+%. ×!"#$%&$' $&$)*# ,!)*%!0 +%.
!+0)1."$% !2 $&$)*#

For studies reporting the same number of patients in both LDLT and DDLT groups or

>/&/04 90/@)./(@.*)()0*3 ℎ 0*2 # 90/@)./(@. &8)+)./(× # &8 × #
#&8 90/)./ (@.*)()0*3 ℎ 0*2 + #&8 90/@)./(@.3&./*&4 0*2
! − # =× (7 (3&*) 8&* 9 +04:): 2)

Variance was calculated as "= *!*+0 #
!2 $&$)*#(#
!2
4+*5$)*×# !2
4+*5$)* 5)
%$#$+%, 5)
%$#$+%,- +%.7-#
+ !2%.
4+*5$)×)1."$%*#
5) !2
,!)*%!0 4+*5$)*
+%. 5)
),!)*%!0! +%.

for studies with a different number of patients in LDLT and DDLT groups.

For example, in the study by Kehar et al., number of deaths at 1-year post-LT was 4 in the LDLT group out of 135 patients and 12 in the DDLT group out of 158, thus the resulting variance is 3,975. The z-score for the pvalue of 0.02= 2.33 thus the O-E= -4.6.”

A. 1-year Graft Survival for LDLT vs Whole graft

Study or Subgroup	LDLT Events	LDLT Total	SPLIT Events	SPLIT Total	O-E	Variance	Weight	Hazard Ratio Exp[(O-E) / V], Fixed, 95% CI
Darius, 2014	10	203	27	138	-9.76	8.91	13.2%	0.33 [0.17, 0.64]
Montenovo, 2018	40	800	223	1784	-27.2	56.21	83.1%	0.62 [0.47, 0.80]
University of Alberta	5	52	6	29	-1.2	2.53	3.7%	0.62 [0.18, 2.13]
**Total (95% CI)**		**1055**		**1951**			**100.0%**	**0.57 [0.45, 0.72]**
Total events	55		256					

Heterogeneity: Chi² = 2.90, df = 2 (P = 0.23); I² = 31%
Test for overall effect: Z = 4.64 (P < 0.00001)

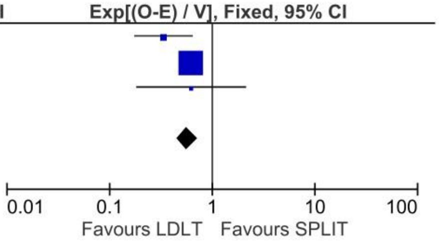

B. 1-year Graft Survival for LDLT vs Reduced deceased graft

Study or Subgroup	LDLT Events	LDLT Total	Whole Events	Whole Total	O-E	Variance	Weight	Hazard Ratio Exp[(O-E) / V], Fixed, 95% CI
Darius, 2014	10	203	10	88	-6.72	4.22	5.8%	0.20 [0.08, 0.53]
Montenovo, 2018	40	800	411	3733	-29.4	65.55	90.7%	0.64 [0.50, 0.81]
University of Alberta	5	52	6	29	-1.27	2.53	3.5%	0.61 [0.18, 2.08]
**Total (95% CI)**		**1055**		**3850**			**100.0%**	**0.60 [0.47, 0.75]**
Total events	55		427					

Heterogeneity: Chi² = 5.19, df = 2 (P = 0.07); I² = 61%
Test for overall effect: Z = 4.40 (P < 0.0001)

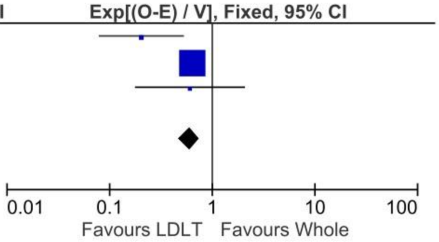

C. 5-year Graft Survival for LDLT vs Whole graft

Study or Subgroup	LDLT Events	LDLT Total	Whole Events	Whole Total	O-E	Variance	Weight	Hazard Ratio Exp[(O-E) / V], Fixed, 95% CI
Darius, 2014	18	203	11	88	-8.09	6.12	5.4%	0.27 [0.12, 0.59]
Montenovo, 2018	64	800	646	3733	-36.9	103.2	91.9%	0.70 [0.58, 0.85]
University of Alberta	6	52	7	29	-1.38	3	2.7%	0.63 [0.20, 1.96]
**Total (95% CI)**		**1055**		**3850**			**100.0%**	**0.66 [0.55, 0.80]**
Total events	88		664					

Heterogeneity: Chi² = 5.38, df = 2 (P = 0.07); I² = 63%
Test for overall effect: Z = 4.38 (P < 0.0001)

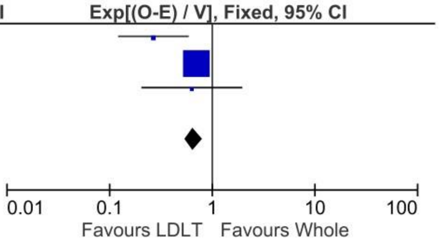

D. 5-year Graft Survival for LDLT vs Reduced deceased graft

Study or Subgroup	LDLT Events	LDLT Total	SPLIT Events	SPLIT Total	O-E	Variance	Weight	Hazard Ratio Exp[(O-E) / V], Fixed, 95% CI
Darius, 2014	18	203	32	138	-11.35	12.05	11.8%	0.39 [0.22, 0.69]
Montenovo, 2018	64	800	346	1784	-33.98	87.6	85.5%	0.68 [0.55, 0.84]
University of Alberta	6	52	6	29	-1.33	2.76	2.7%	0.62 [0.19, 2.01]
**Total (95% CI)**		**1055**		**1951**			**100.0%**	**0.63 [0.52, 0.77]**
Total events	88		384					

Heterogeneity: Chi² = 3.25, df = 2 (P = 0.20); I² = 39%
Test for overall effect: Z = 4.61 (P < 0.00001)

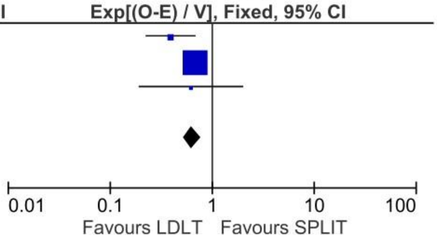

Supplemental Figure S1: Comparison of graft survival stratified by deceased donor graft type

A. 1-year Patient Survival

Study or Subgroup	LDLT Events	LDLT Total	DDLT Events	DDLT Total	O-E	Variance	Weight	Hazard Ratio Exp[(O-E) / V], Fixed, 95% CI	Year
Aydogdu,2005	6	31	4	30	2.9	2.5	6.5%	3.19 [0.92, 11.02]	2005
Oliveros, 2005	1	27	0	27	1.04	0.33	0.9%	23.37 [0.77, 708.66]	2005
Chan,2009	6	59	2	19	-0.68	1.47	3.8%	0.63 [0.13, 3.17]	2009
Zhou,2010	25	201	35	110	-13.59	14.58	37.7%	0.39 [0.24, 0.66]	2010
Darius, 2014	4	203	18	226	-3.466	5.48	14.2%	0.53 [0.23, 1.23]	2014
Tannuri,2016	7	29	11	22	-4.893	4.28	11.1%	0.32 [0.12, 0.82]	2016
University of Alberta	4	52	7	58	-1.726	2.74	7.1%	0.53 [0.16, 1.74]	2018
Szymczak,2018	4	24	10	39	-1.544	3.3	8.5%	0.63 [0.21, 1.84]	2018
Kehar, 2019	4	135	12	158	-4.6	3.975	10.3%	0.31 [0.12, 0.84]	2019
**Total (95% CI)**		**761**		**689**			**100.0%**	**0.50 [0.37, 0.69]**	
Total events	61		99						

Heterogeneity: Chi² = 16.29, df = 8 (P = 0.04); I² = 51%
Test for overall effect: Z = 4.27 (P < 0.0001)

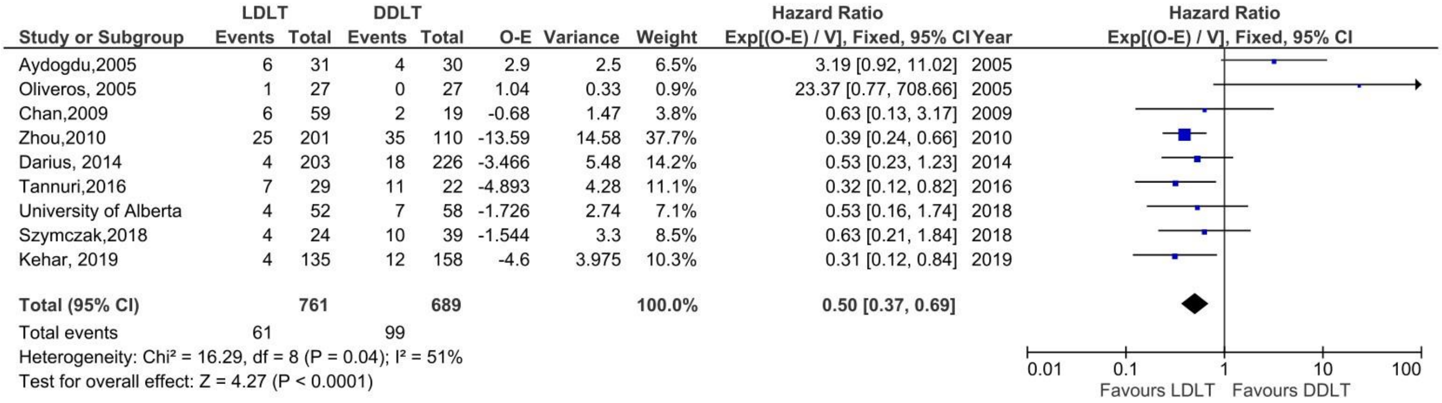

B. 3-year Patient Survival

Study or Subgroup	LDLT Events	LDLT Total	DDLT Events	DDLT Total	O-E	Variance	Weight	Hazard Ratio Exp[(O-E) / V], Fixed, 95% CI	Year
Aydogdu,2005	10	31	9	30	3.94	4.75	13.4%	2.29 [0.93, 5.63]	2005
Chan,2009	6	59	2	19	-0.67	1.47	4.1%	0.63 [0.13, 3.19]	2009
Zhou,2010	31	201	47	110	-15.32	17.83	50.1%	0.42 [0.27, 0.67]	2010
Szymczak,2018	4	24	10	39	-1.53	3.3	9.3%	0.63 [0.21, 1.85]	2018
University of Alberta	4	52	7	58	-1.726	2.74	7.7%	0.53 [0.16, 1.74]	2018
Kehar, 2019	7	135	15	158	-5.45	5.47	15.4%	0.37 [0.16, 0.85]	2019
**Total (95% CI)**		**502**		**414**			**100.0%**	**0.56 [0.40, 0.77]**	
Total events	62		90						

Heterogeneity: Chi² = 11.85, df = 5 (P = 0.04); I² = 58%
Test for overall effect: Z = 3.48 (P = 0.0005)

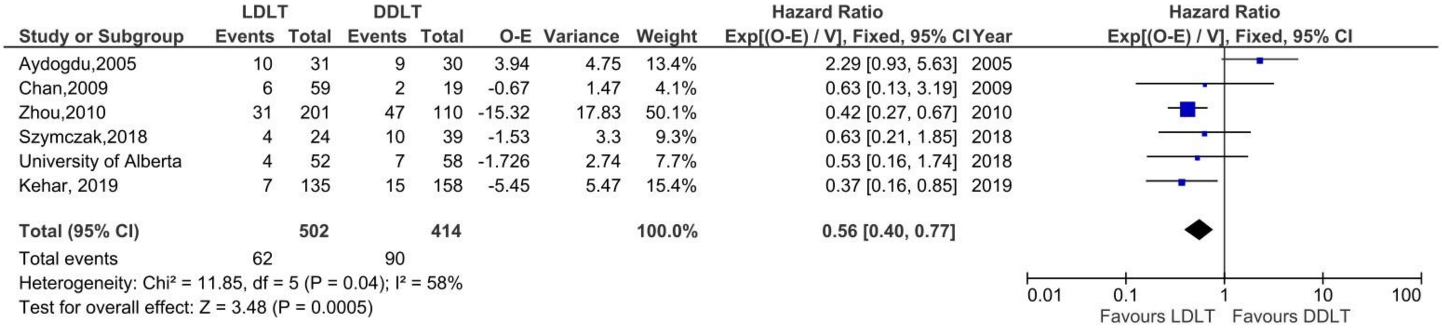

C. 5-year Patient Survival

Study or Subgroup	LDLT Events	LDLT Total	DDLT Events	DDLT Total	O-E	Variance	Weight	Hazard Ratio Exp[(O-E) / V], Fixed, 95% CI	Year
Oliveros, 2005	3	27	1	27	1.64	0.75	2.1%	8.91 [0.93, 85.61]	2005
Darius, 2014	18	203	43	226	-12.75	15.21	42.5%	0.43 [0.26, 0.71]	2014
University of Alberta	6	52	13	58	-3.46	4.74	13.2%	0.48 [0.20, 1.19]	2018
Szymczak,2018	5	24	18	39	-5.05	5.42	15.1%	0.39 [0.17, 0.91]	2018
Kehar, 2019	9	135	30	158	-10.18	9.7	27.1%	0.35 [0.19, 0.66]	2019
**Total (95% CI)**		**441**		**508**			**100.0%**	**0.44 [0.31, 0.60]**	
Total events	41		105						

Heterogeneity: Chi² = 7.40, df = 4 (P = 0.12); I² = 46%
Test for overall effect: Z = 4.98 (P < 0.00001)

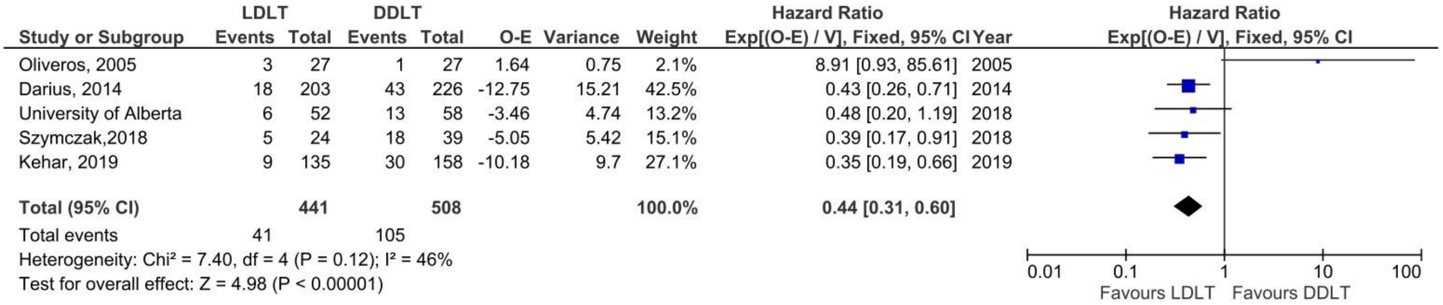

Supplemental Figure S2: Comparison of patient survival between LDLT and DDLT after removal of UNOS data

A. 1-year Graft Survival

Study or Subgroup	LDLT Events	LDLT Total	DDLT Events	DDLT Total	O-E	Variance	Weight	Hazard Ratio Exp[(O-E) / V], Fixed, 95% CI	Year
Oliveros, 2005	3	27	1	27	1.64	0.75	2.5%	8.91 [0.93, 85.61]	2005
Darius, 2014	10	203	37	226	-11.19	11.72	39.7%	0.38 [0.22, 0.68]	2014
Tannuri,2016	2	29	7	22	-3.83	2.2	7.5%	0.18 [0.05, 0.66]	2016
Szymczak,2018	5	24	16	39	-4.83	4.95	16.8%	0.38 [0.16, 0.91]	2018
University of Alberta	5	52	12	58	-3.05	4.2	14.2%	0.48 [0.19, 1.26]	2018
Kehar, 2019	5	135	18	158	-7.84	5.71	19.3%	0.25 [0.11, 0.58]	2019
**Total (95% CI)**		**470**		**530**			**100.0%**	**0.37 [0.26, 0.54]**	
Total events	30		91						

Heterogeneity: Chi² = 9.95, df = 5 (P = 0.08); I² = 50%
Test for overall effect: Z = 5.36 (P < 0.00001)

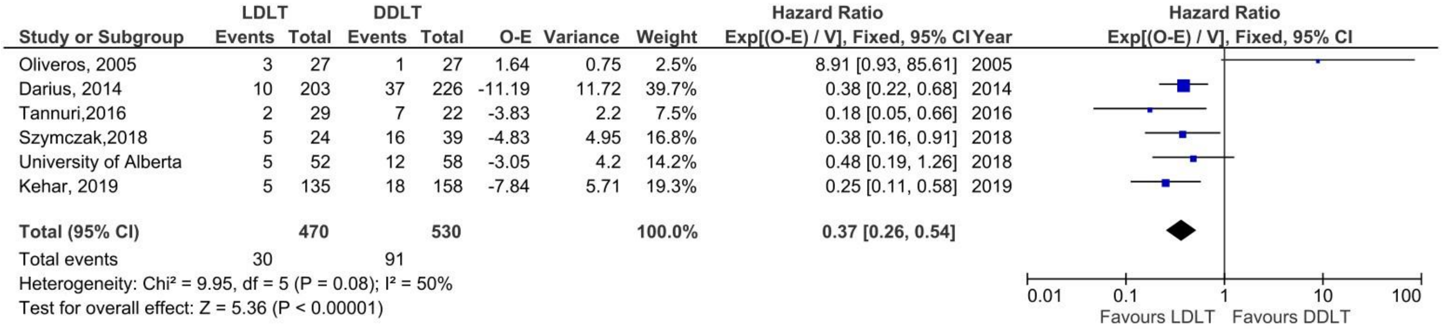

B. 3-year Graft Survival

Study or Subgroup	LDLT Events	LDLT Total	DDLT Events	DDLT Total	O-E	Variance	Weight	Hazard Ratio Exp[(O-E) / V], Fixed, 95% CI	Year
Szymczak,2018	4	24	16	39	-4.7	4.72	26.7%	0.37 [0.15, 0.91]	2018
University of Alberta	6	52	13	58	-3.46	4.74	26.8%	0.48 [0.20, 1.19]	2018
Kehar, 2019	9	135	24	158	-6.21	8.2	46.4%	0.47 [0.24, 0.93]	2019
**Total (95% CI)**		**211**		**255**			**100.0%**	**0.44 [0.28, 0.71]**	
Total events	19		53						

Heterogeneity: Chi² = 0.22, df = 2 (P = 0.90); I² = 0%
Test for overall effect: Z = 3.42 (P = 0.0006)

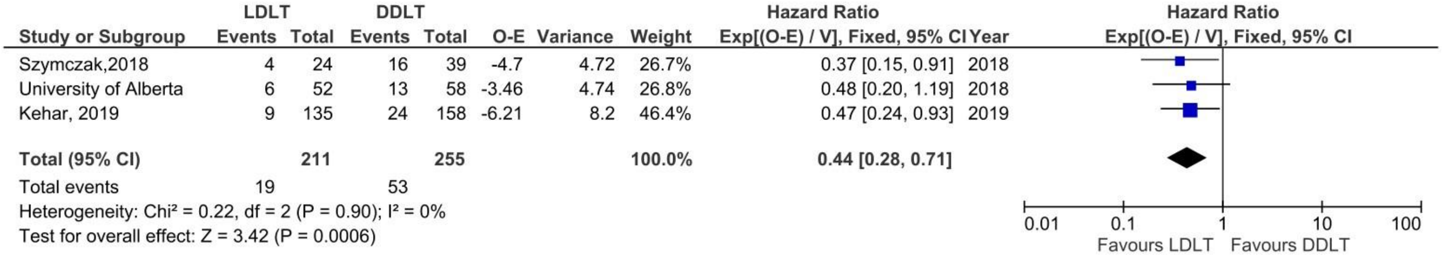

C. 5-year Graft Survival

Study or Subgroup	LDLT Events	LDLT Total	DDLT Events	DDLT Total	O-E	Variance	Weight	Hazard Ratio Exp[(O-E) / V], Fixed, 95% CI	Year
Oliveros, 2005	3	27	1	27	1.64	0.75	2.1%	8.91 [0.93, 85.61]	2005
Darius, 2014	18	203	43	226	-12.75	15.21	42.5%	0.43 [0.26, 0.71]	2014
University of Alberta	6	52	13	58	-3.46	4.74	13.2%	0.48 [0.20, 1.19]	2018
Szymczak,2018	5	24	18	39	-5.05	5.42	15.1%	0.39 [0.17, 0.91]	2018
Kehar, 2019	9	135	30	158	-10.18	9.7	27.1%	0.35 [0.19, 0.66]	2019
**Total (95% CI)**		**441**		**508**			**100.0%**	**0.44 [0.31, 0.60]**	
Total events	41		105						

Heterogeneity: Chi² = 7.40, df = 4 (P = 0.12); I² = 46%
Test for overall effect: Z = 4.98 (P < 0.00001)

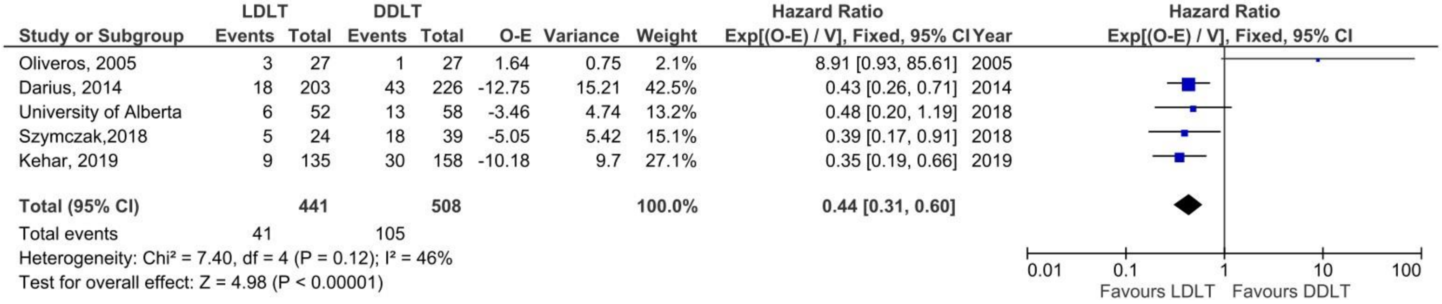

Supplemental Figure S3: Comparison of graft survival between LDLT and DDLT after removal of UNOS data.
